# ASSESSING THE GENERALIZABILITY OF THE PEOPLE LIVING WITH HIV IN THE ALL OF US DATABASE

**DOI:** 10.1093/geroni/igae098.1923

**Published:** 2024-12-31

**Authors:** Narcissa Plummer, Aneeka Ratnayake, Brianne Olivieri-Mui, Robert Cavanaugh

**Affiliations:** Northeastern University, Boston, Massachusetts, United States; Roux Institute at Northeastern University, Portland, Maine, United States; Northeastern University, Boston, Massachusetts, United States; Roux Institute, Northeastern University, Portland, Maine, United States

## Abstract

Few large databases are representative of the US population of people living with HIV. In 2018, the All of Us (AoU) Research Program launched, with the goal of enrolling 1 million Americans under-represented in biomedical research such as PWH. This study aimed to establish the generalizability of data self-reported by PWH in AoU to U.S. Centers for Disease Control and Prevention (CDC) HIV surveillance statistics (2021). We conducted a cross-sectional descriptive analysis of AoU participants who self-reported as living with HIV. The ‘allofus’ R package extracted survey responses to the HIV-related questions with the relevant concept IDs (1384391, 43530505, 43528832, 43530347). We compared AoU demographic statistics to CDC HIV surveillance statistics using Pearson’s chi-squared tests. Of the 409,420 AoU participants 1,714 people self-reported having HIV. Most were male (n=1,282, 76.4%) and reported a non-heterosexual sexual orientation (n=1,239, 71.2%). Almost half were 55 and older (n=821, 47.9%) and the largest race/ethnicity group was non-Hispanic White (n=783, 45.7%). Compared to national surveillance data AoU was similar in gender/sex (US: 76.9% Male vs AoU: 76.4% Male, p-value 0.103), but different in age (US: 22.7% Age 45-54 vs AoU: 26.8% Age 45-54, p-value <0.001) and race/ethnicity (US: 39.8% Non-Hispanic Black vs AoU: 30.6% Non-Hispanic Black, p-value <0.001). AoU has successfully oversampled older ages, but self-reported PWH are Whiter than expected. As such, interpretations of AoU are limited in generalizability to the US HIV population. Future research is to redefine AoU HIV cohort (including health records and medication) and evaluate generalizability.

